# Periodic limb movements during sedation and general anesthesia in elderly patients: a prospective observational study

**DOI:** 10.1007/s10877-026-01421-3

**Published:** 2026-02-24

**Authors:** Katalin Arki, Johannes Harte, Fabienne Frickmann, Darren Hight, Sarah Saxena, Mia Gisselbaek, Anthony R. Absalom, Joana Berger-Estilita, Friedrich Lersch

**Affiliations:** 1 Department of Anesthesiology and Pain Medicine, Inselspital, University Hospital of Bern, Bern, Switzerland; 2 Department of Anesthesiology, Kantonsspital Aarau, Aarau, Switzerland; 3 Department of Anesthesiology, HeloraMons, Belgium; 4 Department of Surgery, Research Institute for Health Sciences and Technology, UMons, University of Mons, Mons, Belgium; 5 Faculty of Medicine, Division of Anesthesiology, Department of Anesthesiology, Clinical Pharmacology, Intensive Care and Emergency Medicine, Geneva University Hospitals, Geneva, Switzerland; 6 Faculty of Medicine, Unit of Development and Research in Medical Education (UDREM), University of Geneva, Geneva, Switzerland; 7 Department of Anesthesia, Montreal Children’s Hospital, McGill University Health Centre, Montreal, QC Canada; 8 Department of Anesthesiology, University of Groningen, University Medical Center Groningen, Groningen, The Netherlands; 9 Institute for Medical Education, University of Bern, Bern, Switzerland; 10 Salem Spital, Hirslanden Medical Group, Bern, Switzerland; 11 Faculty of Medicine, RISE-Health, Centre for Health Technology and Services Research (CINTESIS), University of Porto, Porto, Portugal

**Keywords:** Dexmedetomidine, Periodic limb movements, Actimetry, Processed EEG, Sedation, TAVI, Elderly patients

## Abstract

Dexmedetomidine sedation in elderly patients is associated with cognitive benefits due to its biomimetic non-rapid eye movement (NREM) sleep–like state. However, this state may predispose patients to periodic limb movements (PLMs), which can cause unintended procedural risk during sedation. We aimed to determine the incidence of PLMs during dexmedetomidine-based multimodal sedation for transcatheter aortic valve implantation (TAVI) and explore associations with the need for conversion to general anesthesia. In this prospective observational study, 35 consecutive patients (mean age 81 ± 6 years; 17 female, 18 male) undergoing TAVI over a two-month period from October 2021 to the end of November 2021 were monitored using standard anesthesia monitoring, processed electroencephalography (pEEG) monitoring using Narcotrend 3-lead frontal EEGs (Narcotrend, Hannover, Germany), and bilateral ankle actimetry (SOMNOwatch™ plus). Sedation was administered according to our institutional protocol, beginning with a dexmedetomidine loading dose (0.5 µg kg⁻¹), followed by titration of the infusion between 0.2 and 1.5 µg kg⁻¹ h⁻¹. Additional propofol or fentanyl boluses were administered as clinically indicated by the responsible anesthesiologist. Actimetry, EEG patterns, and intraoperative events were analyzed. PLMs (2–6 movements/min; most commonly 3–5 movements/min) were observed in 20 of 35 patients (57%) and all conversions to general anesthesia (5/5, 100%) occurred in the PLM-positive group, highlighting a potential clinical impact on procedural stability. In the 19 patients with complete EEG datasets, Narcotrend indices during dexmedetomidine-only sedation averaged 96.6 ± 2.6, consistent with an awake–sedate EEG pattern. Following propofol administration, the index decreased to 39.3 ± 20.0, corresponding to a very large effect size (Cohen’s d = 2.85). Five conversions to general anesthesia were necessary in patients with PLMs, because of restlessness, although the severity of PLMs did not predict conversion. No patient had a known history of restless legs syndrome (RLS); prior neurological disease showed no consistent association with PLM occurrence. PLMs are common during dexmedetomidine sedation for TAVI in elderly patients and may need conversion to general anesthesia. While dexmedetomidine offers cognitive benefits, the potential for movement-related procedural risk warrants increased monitoring and consideration during patient selection and anesthetic planning. Further studies comparing sedation-induced and natural sleep PLMs are needed.

## Introduction

Anesthesia and sedation practices for older patients have undergone significant evolution over the past few decades. Growing awareness of postoperative delirium and perioperative cognitive decline has led anesthesiologists to adopt improved neuromonitoring and avoid sedative medications such as benzodiazepines, favoring newer agents such as the α2 agonist dexmedetomidine. Multimodal approaches —combining different anesthetic drugs to maximize therapeutic effect while minimizing adverse effects—and opioid-sparing strategies that include the use of dexmedetomidine have been incorporated into current guidelines and recommendations [1, 2]. Although the evidence base for these approaches continues to develop, they have sparked active debate among clinicians, driven by a shared aim to protect patients from cognitive complications and reduce opioid exposure in the context of the global opioid crisis [3].

While these newer strategies may offer potential benefits, some adverse effects may be overlooked. One such concern is the occurrence of periodic limb movements (PLMs) that have been reported during opioid-sparing anesthesia and sedation in older patients [4]. PLMs are repetitive, stereotyped movements—typically a simultaneous flexion of the foot, knee, and hip—that occur during natural non-rapid eye movement (NREM) sleep. They are particularly common in patients with restless legs syndrome (RLS), as both conditions increase with age and neurodegeneration [5]. In the anesthetic setting, PLMs may arise at critical points in a procedure, such as during transcatheter aortic valve implantation (TAVI) under dexmedetomidine sedation. As TAVI involves manipulation and deployment of rigid guidewires and prosthetic valves within the aorta, even small, unexpected patient movements are considered undesirable and may theoretically increase the risk of malposition or vascular injury; this concern has been one of the arguments in favor of using general anesthesia with neuromuscular blockade to ensure complete immobility during critical phases of the procedure. To date, no study has demonstrated that peri-procedural PLMs during TAVI sedation are associated with increased morbidity or mortality. The concern is currently based on the theoretical risk that abrupt limb or hip movements could interfere with catheter or valve position during critical steps of the procedure [6, 7].

Observational studies and meta-analyses suggest that, in appropriately selected patients, transfemoral TAVI performed under conscious or procedural sedation is at least as safe as, and in some series associated with lower short-term mortality and shorter length of stay than, TAVI performed under general anesthesia [8, 9]. Consequently, many centers favor sedation-based approaches to avoid intubation and deep anesthesia in frail elderly patients, while accepting the trade-off of less controlled immobility. In this context, sedation-associated PLMs represent a potentially relevant, but currently understudied, phenomenon.

The occurrence of PLMs during anesthesia or sedation in older patients remains poorly characterized, despite the potential procedural risks they may pose. This is particularly relevant in the context of multimodal strategies such as dexmedetomidine-based sedation, which—while associated with favorable cognitive outcomes—induces states closely resembling NREM sleep and may therefore predispose patients to PLMs [4]. Current literature offers limited data on how frequently PLMs occur in this setting, which patient populations are most at risk, and whether these movements influence intraoperative management decisions.

To address this knowledge gap, we conducted a prospective observational study to determine the incidence of PLMs during standard-of-care procedural sedation for TAVI. Over a period of two months, we monitored 35 consecutive patients undergoing TAVI to evaluate the frequency of PLMs under various multimodal anesthetic combinations and to assess whether their occurrence necessitated conversion to general anesthesia.

## Objectives

Our primary objective was to confirm the occurrence of PLMs in elderly patients undergoing dexmedetomidine-based procedural sedation for TAVI and to estimate their incidence in this population. The secondary objectives were to estimate the severity of sedation-associated PLMs, expressed using the periodic limb movement index (PLMI), and explore preliminary associations between PLM activity and EEG stages during sedation, acknowledging that the study was not powered for formal inferential analysis.

## Methods

### Ethics statement

The Cantonal Ethics Committee of Bern, Switzerland (KEK Bern; Chairperson Prof. em. Dr. med. Christian Seiler) reviewed the study protocol and waived the requirement for formal ethical approval, as the project involved only the collection and analysis of pseudonymized observational data (Req-2021-01271). Written informed consent was obtained from all participants, or from their legally authorized representatives, for actimetry measurements and the pseudonymized use of their data, in accordance with the Declaration of Helsinki and institutional ethics committee approval.

Identifiable patient information (e.g., date of birth and procedure date) was used only during initial data collection to synchronize recordings and was removed prior to analysis. The final dataset used for evaluation was pseudonymized, with no direct identifiers and no re-identification key available to the analysts.

### Study design and setting

We conducted a prospective observational study at the Inselspital, Bern University Hospital, Switzerland, over a two-month period (October–November 2021). The study included consecutive patients undergoing TAVI for severe aortic valve stenosis under procedural sedation.

### Participants

Patients were eligible if they were scheduled for TAVI under sedation and provided written informed consent for actimetry monitoring and pseudonymized data analysis. The only exclusion criterion was refusal to participate in data collection.

### Standard monitoring

Routine intraoperative monitoring included continuous pulse oximetry, 5-lead electrocardiography, invasive arterial blood pressure monitoring, a triple-lumen central venous catheter, and a 6-French catheter for transvenous pacing placed in the internal jugular vein. Defibrillator pads were positioned on the left and right chest in anticipation of potential hemodynamically significant arrhythmias.

Perioperative neuromonitoring was performed using a three-electrode processed electroencephalography (pEEG) setup and was recorded using the Narcotrend^®^ system (Narcotrend, Hannover, Germany) with a three-electrode frontal montage (Fp1–Fp2–A2). Patients underwent dexmedetomidine-based multimodal sedation, supplemented as needed with propofol boluses or infusion and occasional fentanyl boluses; one patient received a remifentanil infusion with ketamine boluses instead of dexmedetomidine. According to the institutional standard operating procedure (SOP) for TAVI sedation, dexmedetomidine infusion was administered via a syringe pump, initiated with a loading dose of 0.5 µg kg⁻¹ infused over 10 min, and subsequently titrated between 0.2 and 1.5 µg kg⁻¹ h⁻¹ based on clinical requirements. The infusion was typically started shortly before the procedure. Additional sedation was provided with propofol and fentanyl boluses at the discretion of the attending anesthesiologist. Continuous propofol infusion or intravenous ketamine boluses were occasionally administered in cases of inadequate sedation or analgesia, representing permissible deviations from the SOP. Oxygen supplementation was delivered via nasal cannula as standard. Sedation depth was assessed manually and intermittently using the Richmond Agitation–Sedation Scale (RASS) by the attending anesthesiologist at clinically relevant moments rather than at fixed intervals. Clinical assessment included responsiveness to verbal/tactile stimulation, eye opening, respiratory pattern, and hemodynamic changes. Processed EEG monitoring (Narcotrend index, BSR, DSA, and raw traces) was used as an adjunct to support clinical judgement but did not replace bedside assessment. PLMs were identified using a binary classification (movement present/absent) based on synchronized ankle actimetry and direct visual confirmation. No ordinal scale was used by anesthesiologists. PLM severity was quantified retrospectively using the PLMI, calculated as the number of movements per hour of recorded sedation time. Neuromuscular transmission was monitored using standard train-of-four (TOF) stimulation. PLM suppression was confirmed by TOF 0/4, absence of SOMNOwatch actimetry signal, and concomitant deep anesthesia on Narcotrend and BSR metrics.

### Study procedural sedation protocol

All consecutive TAVI patients undergoing procedural sedation were included. Dexmedetomidine was used as the primary sedative agent in 34 of 35 cases. One patient received a remifentanil infusion with rescue ketamine boluses instead of dexmedetomidine due to intraoperative hemodynamic concerns. This patient remained eligible because the study design mandated inclusion of all sedation approaches used in routine practice. Procedural sedation was conducted according to the institutional SOP for TAVI. The protocol steps have been summarized in Table 1.

**Table 1:** Institutional procedural sedation protocol for transfemoral TAVI using dexmedetomidine-based multimodal sedation

Step	Component	Description
1. Initial Sedation	Dexmedetomidine loading dose	0.4–0.5 µg kg⁻¹ over 10 min, shortly before TAVI.
	Infusion titration	Adjusted between 0.2–1.5 µg kg⁻¹ h⁻¹ to achieve moderate sedation with spontaneous breathing.
2. Maintenance Sedation During TAVI	Propofol boluses	10–30 mg IV, repeated as necessary.
	Propofol infusion	0.5–2.0 mg kg⁻¹ h⁻¹ when deeper sedation required.
	Fentanyl boluses	25–50 µg IV for procedural discomfort.
3. Rescue Analgesia / SOP deviations	Ketamine boluses	0.25–0.5 mg kg⁻¹ IV as rescue analgesia.
4. Airway & Oxygen	Supplemental oxygen	2–4 L/min via nasal cannula.
	Capnography	Applied for respiratory monitoring.
	Conversion to GA	Performed if sedation insufficient.
5. Sedation Monitoring	Processed EEG (Narcotrend^®^)	Frontal montage (Fp1–Fp2–A2). Target: calm, arousable (RASS − 2 to − 3).

### Data collection

**Fig. 1:**
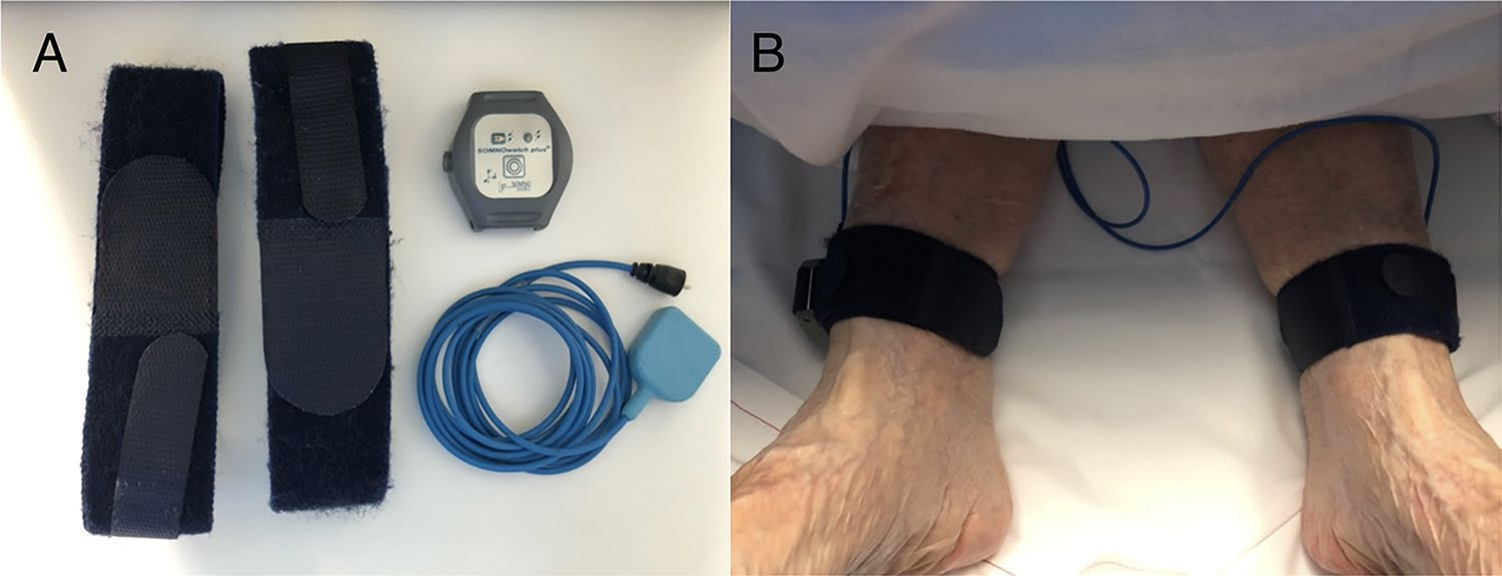
PLM monitoring set up. (**A**) SOMNOwatch™ plus device and its components. (**B**) Application of bilateral ankle actimetry sensors in a TAVI patient

Data were collected prospectively from the initiation of sedation until the end of the procedure. A member of the anesthesia research team was present for all cases to attach SOMNOwatch™ plus actimetry devices (SOMNOmedics GmbH, Randersacker, Germany) to both ankles prior to sedation (Fig. 1A), ensure correct placement of pEEG electrodes, and monitor data acquisition. Devices were secured with textile straps and synchronized with the operating theatre clock to align recordings with procedural events (Fig. 1B).

Actimetry data was stored on-device and downloaded postoperatively using the manufacturer’s software (DOMINO Light, SOMNOmedics). Raw EEG signals and processed pEEG indices (including spectral edge frequency, burst suppression ratio, and a proprietary index) were continuously recorded and exported for offline analysis using the Narcotrend system (Narcotrend Group, Hannover, Germany). Raw EEG signals were not uniformly available for all patients due to intermittent device storage limitations; therefore, only processed EEG indices (Narcotrend^®^) were used for quantitative analyses to ensure full comparability across the dataset. For this exploratory pilot study, EEG evaluation was restricted to representative screenshots of raw EEG, Narcotrend Index, and density spectral array (DSA) output to contextualize sedation depth. Detailed time-locked EEG dynamics during PLM episodes (including NCT, SEF, and BSR fluctuations) were not performed, as a full replay analysis exceeds the scope of this dataset. Sedative drug type, dosage, timing, and deviations from the SOP were extracted from the anesthesia chart. Intraoperative events—including visible PLMs, episodes of restlessness, and conversions to general anesthesia (with timing and indication)—were documented in real time by the attending anesthesiologist.

Demographic and baseline clinical data (age, sex, ASA physical status, neurological history) were obtained from preoperative medical records. After each case, completeness of data acquisition was verified by cross-checking device recordings with clinical documentation. To minimize observer bias, PLM identification was based on concordant evidence from actimetry traces and direct observation by the attending anesthesiologist. PLM episodes were included only when both the SOMNOwatch™ actimetry trace demonstrated a PLM-consistent pattern (≥ 4 movements with PLM-standard duration and inter-movement interval) and the attending anesthesiologist visually confirmed repetitive stereotyped limb movements. Brief or isolated movements detected only by actimetry were not counted as PLMs. Discrepancies between observer and actimetry were rare and typically involved low-amplitude movements below clinical detection thresholds. Device calibration and synchronization procedures were standardized for all cases to reduce measurement variability.

### Actimetry and movement analysis

PLMs were assessed using the SOMNOwatch™ plus system (SOMNOmedics GmbH, Germany), which records triaxial accelerometry on one leg and compares it to a simpler uniaxial motion sensor on the contralateral leg to distinguish unilateral PLMs from whole-body movements. The manufacturer’s algorithm identifies candidate PLM events based on signal amplitude thresholds (≥ 350% and ≤ 700% of baseline acceleration) and temporal criteria defined by the AASM: movement duration 0.3–10 s and inter-movement interval 5–90 s.

**Fig. 2:**
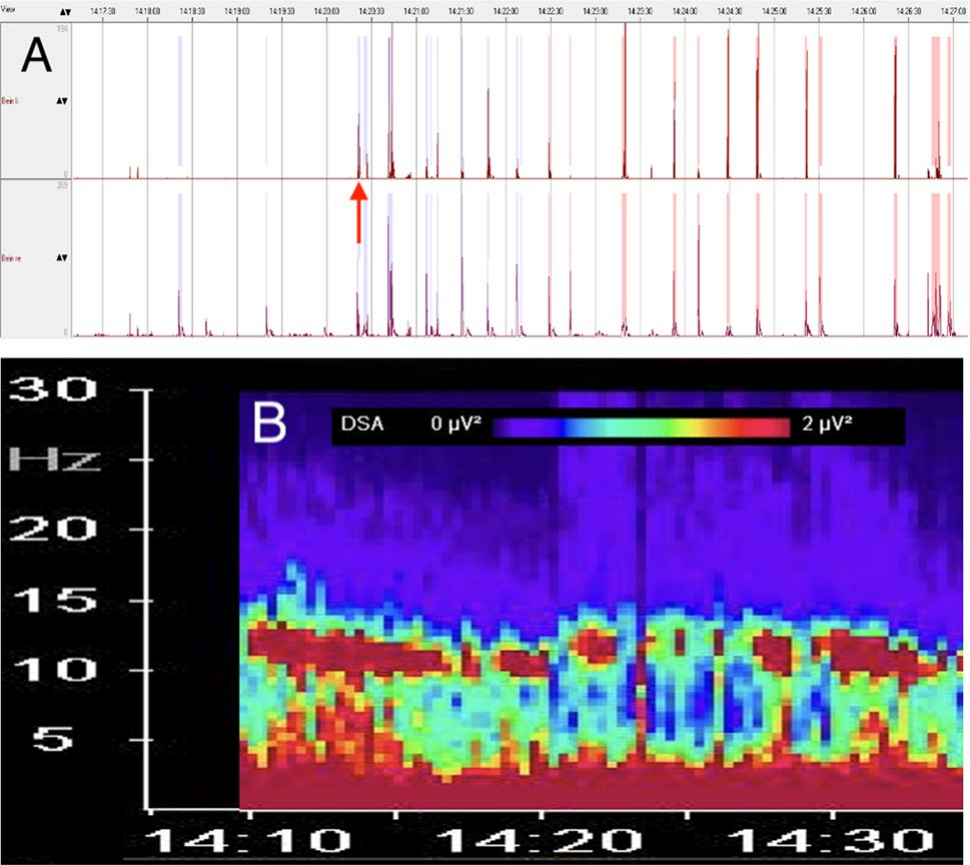
(**A**) The panel illustrates actigraphy recordings, with the left leg displayed in the upper trace and the right leg in the lower trace. The x-axis denotes time, while the y-axis represents actigraphy intensity (µV). Red spikes represent movements fulfilling the PLM periodicity criteria, and blue spikes represent movements that exceed acceleration thresholds but fail periodicity constraints. PLMs commenced at approximately 14:20 (marked with a red arrow) with amplitudes reaching up to 200 µV and a frequency of about three movements per minute. Approximately 40 min earlier, a dexmedetomidine infusion had been initiated, beginning with a bolus of 0.5 µg kg⁻¹ administered over 10 min, followed by a continuous infusion at 0.4 µg kg⁻¹ h⁻¹. Around 20 min before the onset of limb movements, a propofol infusion was started at 60 mg h⁻¹ and subsequently titrated up to approximately 180 mg h⁻¹. (**B**) The panel shows the EEG density spectral array (DSA), with time on the x-axis, frequency on the y-axis, and color intensity representing EEG power (µV²). This example demonstrates the transition from dexmedetomidine sedation to propofol-induced general anesthesia. In the 5 intubated patients the BSR increased to a mean of 28% ± 9%, consistent with deep general anesthesia

In the SOMNOwatch output, red spikes represent movements fulfilling the PLM periodicity criteria, and blue spikes represent movements that exceed acceleration thresholds but fail periodicity constraints (see Fig. 2A). The algorithm does not fully eliminate artifacts; therefore, all automatically flagged events were visually inspected and verified by the investigators.

Raw accelerometry waveforms and algorithm-flagged markers were exported and time-synchronized with Narcotrend EEG using shared timestamps and procedural fiducials, enabling precise matching of PLM episodes to simultaneous EEG state transitions.

#### PLM assessment and PLMI calculation

PLMs were defined according to the American Academy of Sleep Medicine (AASM) scoring rules as stereotyped limb movements lasting 0.5–10 s, occurring in a series of ≥ 4 movements, separated by 5–90 s. The PLMI was calculated as the number of PLM events per hour of analyzed recording time, representing the standard measure of PLM severity used in sleep medicine [10].

PLMI was derived from synchronized SOMNOwatch actimetry recordings after visual verification of each automatically flagged movement event. PLMI values were subsequently used for descriptive and exploratory analyses in this study.

#### PLMI severity classification

In this study, PLM severity refers exclusively to the frequency of PLMs, quantified by the PLMI, defined as the number of PLM events per hour of recorded sedation time. No additional clinical severity dimensions (e.g., arousals, discomfort) were assessed in this intraoperative setting. Severity categories:

- Normal / none: PLMI < 5 events h⁻¹.
- Mild: PLMI 5–24 events h⁻¹.
- Moderate: PLMI 25–49 events h⁻¹.
- Severe: PLMI ≥ 50 events h⁻¹.

This classification is based on standard cutoff values reported in the AASM scoring guidance and widely used in clinical sleep research .

#### Definition of persistent movement

A persistent movement was defined as the occurrence of ≥ 4 PLMs while the patient was within the targeted sedation depth (Narcotrend Index 50–70). This operational definition reflects PLMs that continue despite adequate dexmedetomidine sedation and/or propofol bolus administration, as judged by the attending anesthesiologist and accordance with procedural sedation practice guidelines. This judgment reflected clinically relevant non-response to sedation rather than a predefined PLMI threshold. Persistent restlessness was documented contemporaneously in the anesthesia record whenever additional sedative interventions or conversion to general anesthesia were deemed necessary.

### Study outcomes

The primary outcome was the incidence of PLMs during sedation. PLMs are defined as repetitive, stereotyped flexion movements of the ankle, knee, or hip, occurring in series of at least four consecutive movements lasting 0.5–10 s with inter-movement intervals of 5–90 s [5] PLMs were detected by actimetry and quantified using the PLMI, calculated as the number of movements per hour. Secondary outcomes included (i) conversion from light to deep sedation or to general anesthesia with endotracheal intubation as deemed necessary by the treating anesthesiologist, (ii) type and cumulative dose of sedative and analgesic agents administered, and (iii) the association of PLMs with pre-existing neurological conditions.

### Study size

This was an exploratory pilot investigation. No formal convenience sample size calculation was performed, as no prior data was available regarding the expected incidence of PLMs in patients undergoing TAVI sedation. Therefore, we pragmatically included all eligible and consenting patients during the two-month recruitment period. This approach maximized case capture, ensured feasibility without disruption to clinical workflow, and provided preliminary incidence estimates to inform future hypothesis-driven studies.

### Statistical analysis

Given the exploratory design and small sample size, analyses were primarily descriptive. Continuous variables were summarized as mean ± SD when normally distributed and as median [IQR] when distributions were non-normal. Group comparisons between patients with and without PLMs were performed using the Mann–Whitney U test for continuous variables and Fisher’s exact test for categorical variables. Effect sizes were calculated as rank-biserial correlation for non-parametric comparisons, phi coefficient for categorical data, and Cohen’s d for paired EEG pre/post propofol comparisons. All p-values are interpreted cautiously, as the study was not powered for formal hypothesis testing. A two-sided p value < 0.05 was considered statistically significant. Statistical analyses were conducted using SPSS version 27.0 (IBM Corp., Armonk, NY, USA).

## Results

### Study population

During the two-month study period, 35 consecutive patients undergoing TAVI were enrolled. All participants provided informed consent for actimetry monitoring and data analysis, and no patients were excluded. The cohort consisted of 17 women and 18 men, with a mean age of 81 years (SD, 6; range, 69–95). The baseline comorbidity burden was high, with 20 patients classified as having ASA Physical Status IV and 15 as ASA Physical Status III (Table 2).

**Table 2:** Patient demographics

	Overall	PLM	No PLM
Sex (w./m.)	17/18	9/11	8/7
Age (years)	81.3 (SD 6)	81.3 (SD 6.5)	81.1 (SD 5.4)
Weight (kg)	76.9 (SD 18.4)	79.1 (SD 21)	73.9 (SD 14.4)
Height (cm)	167.1 (SD 9.4)	166.7 (SD 8.3)	167.6 (SD 11)
ASA classification (n)	ASA IV (25), ASA III (10)	ASA IV (15), ASA III (5)	ASA IV (10), ASA III (5)
Neurologic history (n)	13/35	7/20	6/15

None of the patients had a documented diagnosis of restless legs syndrome. Among patients with PLMs, neurological comorbidities included prior brain hemorrhage (*n* = 2), asymptomatic cerebrovascular disease (*n* = 2), Alzheimer’s disease (*n* = 1), migraines (*n* = 1), and head tremor noted preoperatively (*n* = 1).

Patients without PLMs included those with a history of transient ischemic attack (*n* = 3), mild dementia of unknown etiology (*n* = 1), multi-infarct dementia (*n* = 1), and extrapyramidal symptoms (*n* = 1).

### Sedation characteristics

Thirty-four patients received a weight-adjusted dexmedetomidine infusion as per protocol. One patient received no dexmedetomidine as per discretion of the anesthesiologist and instead received a remifentanil infusion with ketamine boluses. The primary clinical objective during vascular cannulation was to achieve mild sedation and attenuate procedural stress while avoiding excessive somnolence. Accordingly, immediately prior to initiation of the TAVI procedure, patients received a dexmedetomidine loading dose of 0.5 µg kg⁻¹ administered over 10 min. The mean initial maintenance infusion rate was 0.48 µg kg⁻¹ h⁻¹ (*n* = 34; range 0.3–0.8 µg kg⁻¹ h⁻¹) and was titrated to a mean maximum of 0.82 µg kg⁻¹ h⁻¹ (*n* = 34; range 0.4–1.5 µg kg⁻¹ h⁻¹). The mean duration of dexmedetomidine administration was 66.7 min (*n* = 34; range 31–102 min).

Additionally, 31 of 35 patients received propofol, either as a continuous infusion (*n* = 6) or as repeated boluses (*n* = 25). Propofol was administered at the discretion of the treating anesthesiologists after increasing dexmedetomidine perfusion. The mean cumulative propofol dose was 85.4 mg (*n* = 31; range 20–330 mg) over a mean procedure duration of 92.4 min (*n* = 35; range 55–129 min, SD ± 17.73). Propofol was administered to reduce agitation and restlessness. Restlessness was assessed clinically by the attending anesthesiologist and defined as visible movement that interfered with procedural stability, necessitating additional intervention (supplemental sedation or conversion to general anesthesia). No additional agitation scale was applied, as real-time motor suppression is the primary adequacy-of-sedation endpoint during TAVI under monitored anesthesia care.

Fentanyl boluses were administered in a minority of cases (8/35 patients), with a mean dose of 15.7 µg (range 25–100 µg). Ketamine (0.25–0.5 mg kg⁻¹ IV) was permitted as a rescue agent for breakthrough movement or discomfort in all patients. In this cohort, only one patient required ketamine supplementation. All sedative regimens and dosing adjustments were made at the discretion of the attending anesthesiologist, in accordance with institutional SOPs. The decision to convert to GA was based solely on clinical findings, mostly confusion and restlessness that rendered the cardiologists’ efforts to place guidewires, balloons and the aortic valve highly risky.

In the 19 patients with complete EEG datasets, Narcotrend indices during dexmedetomidine-only sedation averaged 96.6 ± 2.6, consistent with an awake–sedate EEG pattern. Following propofol administration, the index decreased to 39.3 ± 20.0, corresponding to a very large effect size (Cohen’s d = 2.85). Although raw EEG traces were recorded, they were not consistently available for export across all 19 patients due to device storage limitations and occasional technical interruptions during data acquisition. As a result, only a subset of cases contained complete and usable raw EEG segments.

In one exemplary case, additional insights were obtained from simultaneous actigraphy and EEG spectral analyses. Actigraphy (Fig. 2A) revealed the onset of PLMs at approximately 14:27, with amplitudes reaching 200 µV and a frequency of roughly three events per minute. These movements occurred after administration of dexmedetomidine (0.5 µg kg⁻¹ bolus over 10 min, followed by 0.4 µg kg⁻¹ h⁻¹) and propofol (initiated at 60 mg h⁻¹ and titrated to ~ 180 mg h⁻¹). Concurrently, the DSA (Fig. 2B) demonstrated an alpha-delta spectral pattern that was temporally correlated with the occurrence of the limb movements. The pattern is an alpha-delta dominance during sedation with dexmedetomidine and propofol. Based on the density spectral array (DSA; Fig. 2B), the EEG during dexmedetomidine and propofol sedation exhibited a stable alpha–delta–dominant pattern, consistent with a steady-state level of sedation. Importantly, no EEG features indicative of cortical arousal were observed at the time of PLMs.

### Primary outcome: incidence of PLMs

PLMs were observed in 57% of patients, and all conversions to general anesthesia (5/5, 100%) occurred in the PLM-positive group, highlighting a potential clinical impact on procedural stability. PLMs typically emerged during dexmedetomidine-based sedation within the intended Narcotrend target range (NI 50–70). After propofol supplementation, Narcotrend indices decreased substantially, indicating transition toward deeper anesthesia; however, once triggered, PLMs continued to occur across a wide spectrum of sedation depths and were frequently aggravated following propofol administration. The sedative and analgesic regimens administered, stratified by the presence or absence of PLMs, are summarized in Table 3. Exploratory comparisons between PLM and non-PLM patients are presented with corresponding effect sizes (rank-biserial correlation for continuous variables; phi coefficient for categorical variables), acknowledging that statistical testing is descriptive given the pilot nature of the study. Dexmedetomidine was the primary sedative agent and was administered to 34 out of 35 patients. Doses administered were similar in those with and without PLMs. Propofol was used more frequently and at higher cumulative doses in patients with PLMs (19/20; mean 45 mg). Fentanyl boluses were likewise more common in the PLM group (5/20 vs. 1/15). Persistent restlessness was observed only in PLM-positive patients (5/20, 25%) and was the primary driver for conversion to general anesthesia when present.

Based on the PLMI (number of limb movements per hour of total sedation time), activity was classified as light in 11 patients, moderate in 5 patients, and severe in 4 patients. An elevated PLMI was defined as > 5 events per hour. The light group demonstrated a mean PLMI of 13.2 events/hour (*n* = 11; range 6.6–20.4), the moderate group a mean PLMI of 48.2 events/hour (*n* = 5; range 26.9–64.3), and the severe group a mean PLMI of 97.6 events/hour (*n* = 4; range 67.3–136.5). PLM frequency ranged from 2 to 6 movements per minute, with most episodes in the 3–5 movements/minute range. During PLM episodes, processed EEG indices were approximately 40, corresponding to alpha–delta activity on the raw EEG and spectrogram. All clinically relevant PLM episodes identified by actimetry occurred during periods of stable EEG activity, with no concomitant EEG changes indicative of altered depth of sedation. In particular, no EEG features suggestive of cortical arousal were observed during PLM events. No clinically relevant PLM episode confirmed on actimetry was missed by visual inspection or discordant with concurrent EEG state changes.

**Table 3:** Sedative and analgesic use in patients with and without PLMs

Variable	Overall	PLM (*n* = 20)	Non-PLM (*n* = 15)	Effect size	*p*-value
Dexmedetomidine infusion: N (mean ± SD)	34/35 (51 ± 33 mcg)	20/20 (50 ± 30 mcg)	14/15 (51 ± 37 mcg)	d = -0.03	0.93*
Remifentanil infusion	1/35	0/20	1/15	–	–
Propofol infusion	6/35	4/20	2/15	φ = 0.09	0.68**
Propofol bolus: N (mean ± SD)	31/35 (37.9 ± 26.3 mg)	19/20 (45 ± 30 mg)	12/15 (28 ± 17 mg)	d = 0.67	0.04*
Fentanyl bolus: N (mean ± SD)	6/35 (10 ± 24.4 mcg)	5/20 (13.8 ± 27.5 mcg)	1/15 (5 ± 19.4 mcg)	d = 0.36, φ = 0.24	0.28*/**
Intubation & general anesthesia	5/35	5/20	0/15	φ = 0.35	0.057**

Continuous variables were compared using Welch’s t-test*, with Cohen’s d reported as effect size. Categorical variables were compared using Fisher’s exact test**, with phi (φ) reported as the effect size. Given the exploratory nature of this pilot study, p-values should be interpreted cautiously. Moderate effect sizes were observed for propofol bolus dosing (d = 0.67) and conversion to general anesthesia (φ = 0.35), while all other differences showed small or negligible effects

### Secondary outcomes

#### Conversion to general anesthesia

Sedation was converted to general anesthesia with endotracheal intubation in 5 patients (14%), all of whom displayed PLMs. Conversions were necessitated by persistent restlessness rather than the severity of PLM activity, as patients with higher PLMI values did not uniformly require intubation. Following induction and intubation, burst suppression was consistently observed. Importantly, PLMs continued until complete neuromuscular blockade was established (TOF 0/4). After paralysis, SOMNOwatch signals flattened and BSR increased significantly (mean 28% ± 9%), confirming both immobility and deep hypnotic state.

## Discussion

In this prospective observational study, we found that PLMs occurred in more than half of older patients undergoing TAVI in dexmedetomidine-based sedation. This is, to our knowledge, the first study to quantify PLMs in this context using both actimetry and processed EEG monitoring. While dexmedetomidine is increasingly utilized in multimodal sedation and general anesthesia—inducing a state often described as “biomimetic sleep”—it is important not to overlook potential adverse effects, such as postoperative myoclonus, even as we aim to utilize its cognitive benefits for elderly patients [11]. Perioperative PLMs may share mechanistic features with postoperative myoclonus, including disinhibition of spinal locomotor pattern generators during reduced cortical input. Dexmedetomidine and propofol can both paradoxically facilitate excitatory motor phenomena despite profound sedation, supporting a subcortical or spinal locus of motor generation rather than a purely cortical mechanism [12–14]. The large effect size underscores a robust transition in EEG activation state after propofol administration, supporting physiological plausibility despite the exploratory nature of this pilot dataset. Moreover, our data indicates that periodic PLMs are not linked to inadequate sedation or anesthesia depth. We observed persistent PLM activity in patients who were deeply sedated, exhibiting alpha-delta EEG patterns or even burst suppression. Clinicians should recognize that involuntary PLMs can occur in elderly patients under adequate or even “deep anesthesia”, and these movements should not be interpreted as a sign that additional sedative dosing is required [15]. This population is already at risk of being over-sedated even when monitored with EEG [11, 16]. The observed aggravation of PLMs following propofol supplementation is consistent with propofol’s known excitatory effects on spinal locomotor circuitry and its potential to disinhibit subcortical motor pattern generators, even during deep hypnosis. Once triggered, PLMs may therefore persist or intensify despite increasing hypnotic depth, highlighting that cortical suppression alone does not abolish this motor phenomenon. Increased dosing of hypnotics based on observed PLMs that are interpreted as inadequate depth of anesthesia entails longer and greater time-windows of burst-suppression with potentially adverse cognitive consequences for elderly patients [17]. No burst suppression activity was present during dexmedetomidine-based procedural sedation. Five of 35 patients (14%) required conversion to general anesthesia due to persistent restlessness, and all of them belonged to the PLM-positive group (100%). Following propofol induction and neuromuscular blockade, burst suppression was present with a mean BSR of 28 ± 9%, consistent with profound hypnotic depth during intubation. Persistent PLMs ceased immediately after neuromuscular blockade.

We have previously reported PLM activity in a patient undergoing eye surgery initially under opioid-free GA without muscle relaxation [18]. PLMs were present during general anesthesia characterized by alpha-delta or even burst suppression EEG activity. The persistence of PLMs across variable EEG sedation states suggests that their generation is not directly determined by cortical hypnotic depth. Rather, PLMs may represent a spinal or subcortical motor phenomenon that, once initiated, remains active despite deeper anesthesia levels.

PLMs are generated by central pattern generators (CPGs) located in the spinal cord, which produce rhythmic motor output independent of voluntary control. The excitability and activity of these CPGs are modulated by descending pathways from the brain, notably dopaminergic, opioidergic, and noradrenergic systems [19]. The underlying mechanism of PLMs appears to be caused by abnormal excitability within the spinal cord, in particular in the lumbosacral segments. The exact nature of supraspinal influence remains incompletely understood. Neurophysiological studies show that PLMs are characterized by repetitive, stereotyped muscle contractions, most frequently beginning in the tibialis anterior muscle. The contractions often alternate between limbs without a consistent recruitment pattern, indicating the presence of multiple, independent, and unsynchronized spinal generators, rather than a single centralized motor source [20].

Moreover, reports of PLMs in awake patients under spinal or epidural anesthesia further underline the possibility of disinhibition of CPGs in the spinal cord [21, 22]. CPGs may be regulated by striatal and thalamic control centers. Evidence for this comes from post-mortem histological examinations of these areas in patients who were dopamine-deficient and suffering from RLS. Further evidence comes from murine studies, where impaired activity in local spinal interneurons appears to disinhibit CPGs [23]. Dexmedetomidine is believed to primarily modulate CPGs and PLMs through its effects on descending noradrenergic pathways, rather than direct modulation of dopaminergic or opioidergic descending input [24]. Dexmedetomidine has been shown to increase the activity of ventral tegmental area dopamine neurons and elevate dopamine transmission in forebrain projection areas, but this is primarily associated with its sedative and arousal properties rather than direct modulation of motor pattern generators or PLMs [25]. Although PLMs in natural sleep are most associated with NREM2/3 stages, the presence of PLMs during deep anesthesia and even during burst suppression indicates that PLM generation does not depend on a specific sleep-stage EEG structure. Instead, PLMs likely arise from subcortical or spinal pattern generators that become disinhibited when cortical control is reduced—whether through physiological sleep or pharmacological anesthesia.

The occurrence of PLMs during sedation and anesthesia is an under-recognized but potentially important phenomenon. All conversions from sedation to general anesthesia occurred in patients with PLMs, although the frequency or severity of the movements did not predict the need for conversion. Dexmedetomidine produces a sedation pattern resembling non-rapid eye movement (NREM) sleep, which may help protect against postoperative delirium by avoiding the deeper burst suppression states often seen with general anesthesia [21].

The present study addresses the occurrence of periodic limb movements exclusively under procedural sedation and does not allow direct conclusions regarding PLMs during natural sleep. Although previous work suggests that PLMs may emerge under conditions of reduced cortical control, including physiological sleep and pharmacologically induced states, the relationship between sedation-associated PLMs and PLMs occurring during natural sleep remains speculative [26, 27]. However, it may predispose patients to PLMs, particularly the elderly, who are already more prone to these movements during natural sleep [28]. In the setting of TAVI, even small, untimely movements could cause displacement of rigid guidewires or prosthetic valves, with the potential for serious procedural complications. In our study, conversions to general anesthesia appeared to be triggered by overall restlessness rather than the absolute severity of PLMs.

The fact that PLM intensity was not diminished by opioid administration during these sedations is a noteworthy observation. In treating Periodic Limb Movement Disorder, opioids are sometimes prescribed when first-line treatments like iron supplementation and dopamine agonists fail to provide relief [23, 29]. A small study tested ketamine as a rescue treatment [30]. Opioids such as fentanyl can suppress movement by reducing spinal locomotor generator activity [31, 32], whereas NMDA receptor antagonists like ketamine modulate excitatory motor pathways and thereby may attenuate PLMs [33]. These pharmacologic mechanisms may contribute to the reduced PLM expression observed following the initiation of general anesthesia. Further research is needed to examine the incidence of PLMs in various anesthesia and analgesia combinations, particularly in patients with RLS or those undergoing PLM assessment during nocturnal sleep. Such studies are likely to significantly enhance our understanding of the pathophysiology underlying sleep-related disorders.

Our findings contrast with the well-established link between PLMs and RLS in the sleep literature, as none of our patients had a documented diagnosis of RLS. This raises the possibility that PLMs under sedation may occur independently of RLS, perhaps mediated by sedation-induced changes in sleep architecture. While isolated reports have suggested that sedatives and anesthetics can modulate PLM frequency, our study is the first to provide incidence data in elderly patients undergoing a high-risk, catheter-based procedure.

Although dexmedetomidine is not a new sedative agent, the occurrence of PLMs under its use has not been systematically reported in anesthesia practice. Several factors may explain this. First, dexmedetomidine was initially used predominantly in the intensive care setting, where subtle motor activity may have been less apparent or considered clinically insignificant. In the operating room, most sedative regimens historically relied on agents such as benzodiazepines, propofol, or opioids, which suppress motor activity more uniformly and thus mask PLMs.

Second, without dedicated monitoring, PLMs could easily be misinterpreted as agitation, pain, or inadequate sedation, prompting additional hypnotics or opioids rather than recognition of a distinct motor phenomenon. Third, perioperative use of synchronized processed EEG and actimetry has only recently become more widespread, enabling more precise correlation of stereotyped limb activity with specific sedation states.

Finally, PLMs would not be observed in general anesthesia with neuromuscular blockade, which remains the standard for most surgical procedures. It is specifically in non-intubated procedures such as TAVI, where sedation is maintained without muscle relaxation, that these movements become evident and clinically relevant.

Our results have practical implications. Awareness of PLMs could influence sedative choice, titration strategies, and readiness for unplanned conversion to general anesthesia in TAVI procedures. Preoperative screening for sleep movement disorders might be considered, although the predictive value remains uncertain. Given the delicate and potentially hazardous phases of TAVI, a better understanding of PLMs under sedation could help improve patient safety.

As an additional observation, our small study raises important questions about how sedation may serve as a test of resilience of the aging brain, given that only a limited number of patients required intubation due to restlessness and confusion [34]. It remains unclear whether PLMs observed during sedation and general anesthesia are linked to post-procedural outcomes such as delirium or prolonged emergence in elderly patients.

The strengths of our study include its prospective design, synchronized actimetry and pEEG monitoring, and real-time clinical observation by the anesthesia team. All eligible patients included during the study period minimized selection bias.

However, several limitations exist. Firstly, it was a small, single-center pilot study with only 35 patients. The study was intentionally designed as a small, prospective pilot aimed at estimating PLM incidence and characterizing associated EEG patterns rather than testing predefined hypotheses. Accordingly, the statistical analysis serve to contextualize the descriptive observations, and all inferences should be interpreted with appropriate caution. As only five conversions to general anesthesia occurred, regression analyses relating PLMI to conversion were not statistically feasible due to the high risk of overfitting and complete separation bias. These relationships will be examined in our larger, ongoing study. Secondly, complete EEG data were available in just over half of the patients, restricting correlation analyses between sedation depth, PLM activity, and conversion to general anesthesia. Although EEG was collected in most patients, the variable completeness and artifact contamination of raw EEG prevented time-locked statistical analysis. To avoid misleading inference, EEG findings are presented descriptively. Future work with continuous high-quality EEG acquisition will enable more robust mechanistic conclusions. Our ongoing dataset includes complete raw EEG export with synchronized actimetry, enabling detailed characterization of NCT, SEF, BSR and DSA behavior during PLMs, which was not achievable in this pilot study. Third, patients were not systematically screened for RLS or other sleep-related movement disorders, raising the possibility of undiagnosed conditions contributing to PLM incidence. Fourth, PLMs were identified pragmatically using actimetry and clinical observation rather than full polysomnography, which may affect comparability with sleep medicine standards. Finally, sedative regimens beyond dexmedetomidine were not fully standardized, as adjunctive agents were administered at the discretion of the attending anesthesiologist, potentially introducing variability in PLM expression and management. We retained the single patient who did not receive dexmedetomidine because excluding them would introduce selection bias in this consecutive real-world cohort. This patient did not exhibit PLMs, and their inclusion did not affect incidence estimates.

Although the study was not designed as a formal device-validation analysis, concordance between SOMNOwatch actimetry and anesthesiologist observation was high for clinically relevant PLM sequences. No clearly observable PLM episode was missed by actimetry, while the device occasionally detected low-amplitude movements that did not meet full PLM criteria. Future studies employing blinded dual scoring would allow formal estimation of inter-rater and device–observer reliability.

Future studies should aim to replicate our findings in larger and more diverse populations, compare the incidence of PLMs across different sedative regimens, and investigate the potential impact on procedural outcomes. Inquiring into how the presence of PLMs in nightly sleep predicts PLM activity in patients undergoing either sedation or general anesthesia without muscle relaxation may offer valuable insights into how sleep-associated movement disorders resurface during anesthesia. Contrary to current opinion, these phenomena may blur the distinct border between sleep and anesthesia [35]. Further work is also needed to explore EEG patterns during sedation-associated PLMs and to determine whether targeted preoperative screening or specific pharmacological strategies can reduce their occurrence.

## Data Availability

No datasets were generated or analysed during the current study.

## Abbreviations

ASA: American Society of Anesthesiologists
BSR: Burst Suppression Ratio
CPG: Central Pattern Generator
DSA: Density spectral array
Fp: Pre-frontal
NCT: Narcotrend
NREM: Non-rapid eye movement
pEEG: Processed Electroencephalography
PLM: Periodic limb movements
PLMI: Period Limb Movement Index
RLS: Restless leg syndrome
SD: Standard deviation
SEF: Spectral edge frequency
SOP: Standard of Procedure
TAVI: Transcatheter Aortic Valve Implantation
